# Monitoring Prevention Impact of Mother-to-Child Transmission of HIV in Concentrated Epidemics With Program and Survey Data

**DOI:** 10.2196/publichealth.7701

**Published:** 2017-12-20

**Authors:** Thi Thuy Van Nguyen, Keith Sabin, Thi Quynh Trang Ho, Ai Kim Anh Le, Chika Hayashi, Masaya Kato

**Affiliations:** ^1^ World Health Organization Country Office Hanoi Viet Nam; ^2^ Thai Nguyen Provincial AIDS Centre Thai Nguyen Viet Nam; ^3^ World Health Organization HIV/AIDS Geneva Switzerland

**Keywords:** HIV, prevention, mother-to-child transmission, Vietnam

## Abstract

**Background:**

The prevention of mother-to-child transmission (PMTCT) of HIV program was introduced in Vietnam in 2005. Despite the scaling up of PMTCT programs, the rate of mother-to-child HIV transmission in Vietnam was estimated as high as 20% in 2013.

**Objective:**

The objective of this study was to assess the outcomes of PMTCT and identified factors associated with mother-to-child transmission and infant survival using survey and program data in a high HIV burden province in Vietnam.

**Methods:**

This community-based retrospective cohort study observed pregnant women diagnosed with HIV infection in Thai Nguyen province from October 2008 to December 2012. Data were collected through interviews using a structured questionnaire and through reviews of log books and medical charts in antenatal care and HIV clinics. Logistic regression and survival analysis were used to analyze data using Stata (StataCorp).

**Results:**

A total of 172 pregnant women living with HIV were identified between 2008 and 2012. Most of these women had acquired the HIV infection from their husband (77/119, 64.7%). Significant improvement in the PMTCT program was documented, including reduction in late diagnosis of HIV for pregnant women from 62.5% in 2008 to 30% in 2012. Access to antiretrovirals (ARVs) improved, increasing from a rate of 18.2% (2008) to 70.0% (2011) for mothers and from 36.4% (2008) to 93.3% (2012) for infants. For infants, early diagnosis within 2 months of birth reached 66.7% in 2012 compared with 16.7% in 2009. Transmission rate reduced from 27.3% in 2008 to 6.7% in 2012. Late diagnosis was associated with increased risk for HIV transmission (odds ratio [OR] 14.7, 95% CI 1.8-121.4, *P*=.01), whereas ARV therapy for mother and infant in combination with infant formula feeding were associated with reduced risk for HIV transmission (OR 0.01, 95% CI 0.001-0.1; *P*<.001). Overall survival rate for HIV-exposed infants at 12 months was 97.7%.

**Conclusions:**

A combination of program and survey data measured the impact of prevention of HIV transmission from mother-to-child interventions. Significant improvement in access to the interventions was documented in Thai Nguyen province. However, factors that increased the risk of HIV transmission, such as late diagnosis, remain to be addressed.

## Introduction

There are nearly 5 million people living with HIV (PLHIV) in Asia and the Pacific region, and key populations at the greatest risk for contracting HIV are people who inject drugs (PWID), men who have sex with men (MSM), and female sex workers (FSW) [1]. Vietnam is one of the 12 countries with the highest HIV burden in the region [1]. As of 2014, it was estimated that 256,000 people were living with HIV in Vietnam. The HIV prevalence among PWID, FSW, and MSM in 2013 was 22%, 5.3%, and 2.4%, respectively, whereas HIV prevalence in general population and pregnant women was 0.26% and 0.2%, respectively [2]. The prevalence of HIV among pregnant women varies by provinces. For example, it was 0.25% in Thai Nguyen province in 2014 (unpublished data) and higher (0.42%) in Ho Chi Minh City in 2013 [3].

Vietnam introduced the prevention of mother-to-child transmission of HIV (PMTCT) program in 2005. Since then, the proportion of pregnant women who received an HIV test and their results, including those with previously known status as HIV positive, increased from 11% in 2008 to 50% in 2013 [2]. Access to antiretrovirals (ARVs) to reduce the risk of mother-to-child transmission improved concurrently. The proportion of HIV-positive pregnant women receiving ARVs increased from 33% in 2008 to 57% in 2013 [2]. Despite these improvements in access to HIV testing and ARVs for pregnant women, the mother-to-child HIV transmission rates were estimated as high as 20% in 2013 because of limited coverage of PMTCT, late diagnosis, and delayed antiviral intervention [2].

A universal HIV testing policy for pregnant women is not yet adopted by Vietnam. Resources for PMTCT are focused on provinces with high and medium HIV burden as determined by HIV case reporting and mainly supported by donors such as the US President’s Emergency Plan for AIDS (acquired immunodeficiency syndrome) Relief as well as the Global Fund to Fight AIDS, Tuberculosis and Malaria. Currently, because of limited resources, a universal testing policy is only applied in high-burden districts. At the same time, the Ministry of Health is developing a standard package for antenatal care (ANC), which includes HIV, hepatitis B virus, and syphilis testing. Advocacy to cover these tests under health insurance is ongoing.

At the time of this study, ARV prophylaxis using azidothymidine (AZT) was recommended for all HIV-infected pregnant women to prevent transmission to their neonates (option A per WHO [World Health Organization] 2010 guidelines) [4]. A short course of ARV prophylaxis (single dose of nevirapine [NVP] and 4 weeks of AZT) was recommended for HIV-exposed infants. Infant formula was provided free of charge for up to 18 months. The current national guidelines that were updated in 2015 recommend ARV treatment for all pregnant women regardless of cluster of differentiation 4 (CD4) and clinical stage, as well as 6 to 12 weeks of NVP for all HIV-exposed infants [5].

In line with the guidance from WHO and the Joint United Nations Program on HIV and AIDS (UNAIDS), the Vietnam National Strategy on HIV/AIDS Prevention and Control has set a target of reducing HIV transmission from mother to child to less than 2% by 2020 [6], with an aim of eliminating HIV transmission from mother to child by 2015. To reach this target, identification of the barriers to the elimination of mother-to-child transmission is important.

The barriers may exist at any stage of the PMTCT cascade, which includes HIV testing of pregnant women, referral of HIV-positive women for ARV therapy (ART), uptake of ART by pregnant women living with HIV, treatment of neonate, infant feeding, and early infant diagnosis. Data from Vietnam and elsewhere show a range of barriers to access PMTCT services, including individual and health system issues such as lack of knowledge and information, fear of stigma and discrimination, late diagnosis, poor quality of care, and service accessibility [7,8]. Thus, understanding what factors were associated with mother-to-child transmission within the program context would help program managers to design appropriate service delivery models and allocation of resources for optimizing the effectiveness of recommended interventions. With this fact in mind, this study was conducted in Thai Nguyen, a province located in the north of Vietnam with a high HIV prevalence among PWID (34%) [2], which aimed to use a method relying primarily on routine data to assess the outcomes of the PMTCT program in Thai Nguyen and to identify factors associated with HIV transmission from mother to child and infant survival.

Guidelines of UNAIDS and WHO encourage a shift from using ANC survey data to using the program data for HIV. As countries confront the required quality improvement for program data to make this shift, implementers should consider the additional steps that could improve impact monitoring of PMTCT programs at the same time.

This study aims to assess the outcomes of PMTCT and identified factors associated with mother-to-child transmission and infant survival using survey and program data in a high HIV burden province in Vietnam.

## Methods

### Data Collection

This community-based retrospective cohort study was conducted between November 2011 and January 2013 to assess the outcomes of the PMTCT program, including uptake of ANC and PMTCT services among pregnant women living with HIV, infants’ HIV and survival status, and associated factors. A list of pregnant women who were diagnosed with HIV infection in the entire province, including 9 districts and towns, from October 2008 to 2012 was developed. After removing duplicate cases, the list was finalized with 172 pregnant women living with HIV. The records of these women were traced back through registration in outpatient clinics (OPCs) and district PMTCT clinics. All 172 HIV-infected pregnant women were contacted by OPC doctors either through telephone numbers that they had given upon registration or through the peer educators who support the treatment program, and they were invited for a face-to-face interview at their district OPCs or at their preferred locations. For women who were not contactable by telephone, the health care workers at the district PMTCT focal point visited them in their house to invite them to participate in the study. The interviews were conducted with those who provided informed consent by the provincial AIDS center staff using a structured questionnaire. Information on demographics, mothers’ access to HIV testing and ARVs, infant ARV prophylaxis, and infant feeding was collected through the interview.

**Figure 1:**
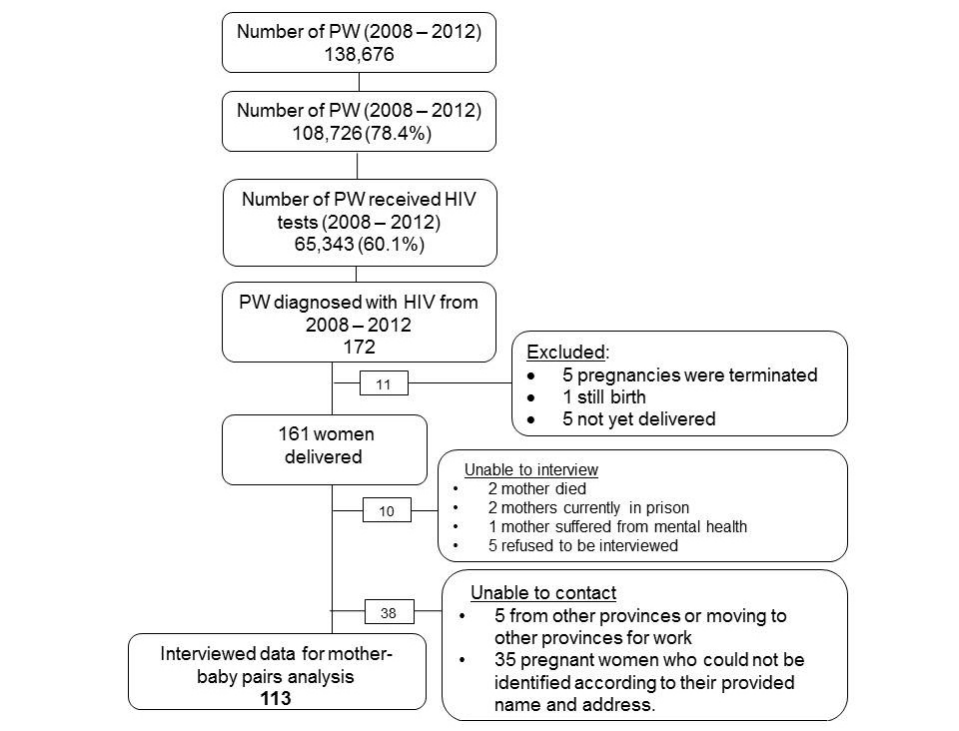
Flow chart of recruiting participants. PW: pregnant women.

Data related to ART for the mothers during labor and pregnancy, access to HIV care, and treatment for mothers and infants were also collected through the review of medical records in provincial and district obstetrics departments, pediatric clinics, and OPCs. Wherever possible, data from interviews were triangulated with data from medical charts to validate interview data.

### Data Analysis

Data collected from interviews and linked to medical records were analyzed to describe characteristics of the pregnant women living with HIV and their access to PMTCT interventions.

To determine the transmission rate and HIV-free survival, data related to this cohort of infants born to HIV-infected mothers were analyzed. The endpoint for the infant follow-up was HIV status as recorded either at the last visit to a pediatric clinic or at the time of the death. Children with unknown HIV status who did not return to a pediatric clinic within 90 days of the last visit were defined as lost to follow-up, and the date of last visit recorded in the medical chart was used as the censoring date. Survival rate was determined among infants with and without HIV infection by survival analysis.

For analysis of HIV transmission risk factors, only 113 mother-infant pairs were available for inclusion in logistic regression analysis. We excluded those who could not be interviewed for various reasons or whose pregnancies were terminated (see Figure 1). To understand the effect of combined interventions of ARVs for mother, ARV prophylaxis for infant, and infant formula feeding, a new variable was generated with three level categories: full access (received all three interventions), partial access (received one or two interventions), and no access (missed all interventions).

Data analysis was performed by using STATA 11.1. Logistic regression and survival analysis were conducted to identify factors associated with HIV transmission from mother to child and survival rate for HIV-exposed infants.

The study was reviewed and approved by Local Ethics Review Committee—Hanoi School of Public Health—and by Ethics Review Committee of WHO Regional Office for Western Pacific.

## Results

From 2008 to 2012, there were 108,726 out of 138,676 (78.40%) pregnant women enrolled for pregnancy management in the province. Of those who enrolled, 65,343 (60.1%) received HIV tests (Figure 1). Prevalence of HIV among pregnant women who received HIV testing was 0.26% (172/65,343). Among 172 HIV-positive pregnant women, 161 women delivered and 113 were interviewed (Figure 1). Most of the interviewed pregnant women living with HIV were young (mean age: 28.2 years) and married (94.1%). More than half (55.5%) had an education level of high school or higher, and 58.0% reported having a stable job. Nearly one-third of the interviewed women were from ethnic minority groups. The most common route of infection reported was from an HIV-positive husband (64.7%). Although less common, nearly 3% of the women reported that they had more than one sexual partner, and 3.4% of the women reported ever sharing needles when injecting drugs (Table 1).

### Uptake of Antenatal Care Among HIV-Positive Women

**Figure 2:**
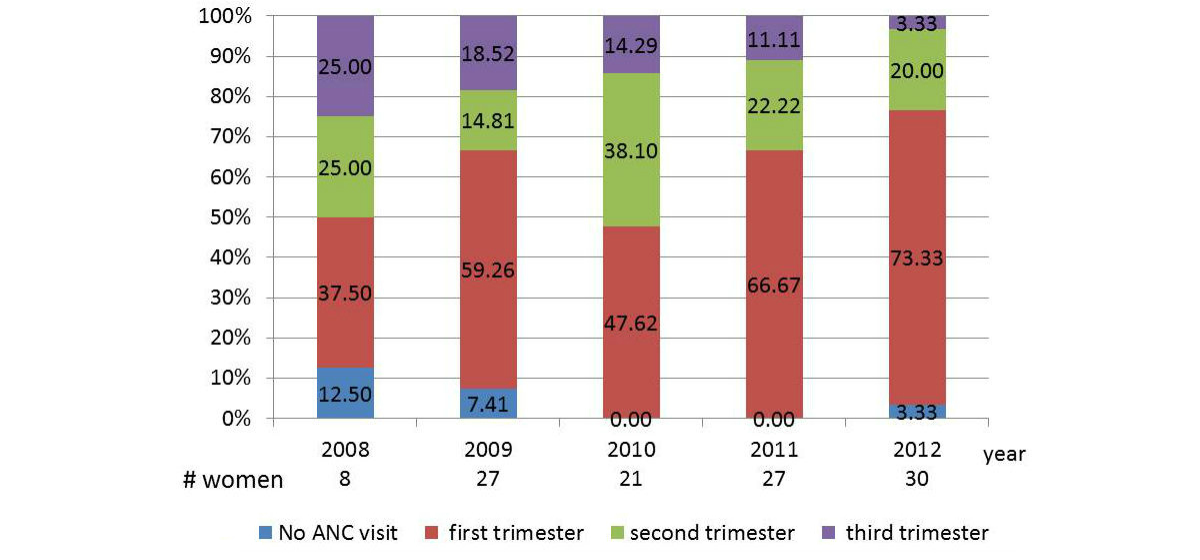
Time of first antenatal care visit among pregnant women living with HIV infection by year. ANC: antenatal care.

The proportion of pregnant women with a first ANC visit during the first trimester improved over the study period, whereas the number of late ANC visits (during trimester) or number of women with no ANC visit declined (Figure 2). A majority of women delivered at national/provincial hospital (76.3%) or district health facilities (19.3%), whereas a small number delivered at community health stations or at home (4.4%). Cesarean sections were 36.8% of all deliveries (Table 1).

**Table 1:** Characteristics of pregnant women living with HIV infection (including 5 women who terminated their pregnancies and 1 woman who had still birth).

Variables		Values (N=119)
Age range in years (mean, standard deviation)		19-42 (28.2, 4.5)
**Marital status, n (%)**		
	Married	112 (94.1)
	Other (single, widow, and divorced)	7 (5.9)
**Ethnicity, n (%)**		
	Kinh	87 (73.1)
	Minority	32 (29.3)
**Education, n (%)**		
	Primary or secondary school	53 (44.5)
	High school or higher	66 (55.5)
**Employment**^a^**, n (%)**		
	Stable job	69 (58.0)
	Unstable job	31 (26.1)
	Unemployed	18 (15.1)
**Reported transmission route, n (%)**		
	Having more than one sexual partners	3 (2.5)
	Spouse infected with HIV	77 (64.7)
	Spouse using drugs and unknown HIV status	21 (17.6)
	Ever injecting drugs and sharing needles	4 (3.4)
	Unknown/No answer	14 (11.8)
**Location of delivery**^b^**, n (%)**		
	District hospital	22 (19.3)
	National/Provincial hospital	87 (76.3)
	Community health station or at home	5 (4.4)
**Mode of delivery**^b^**, n (%)**		
	Natural delivery	72 (63.2)
	Cesarean	42 (36.8)

^a^Value missing for one.

^b^Of the total, 5 women had their pregnancy terminated.

### Access to PMTCT Services

**Figure 3:**
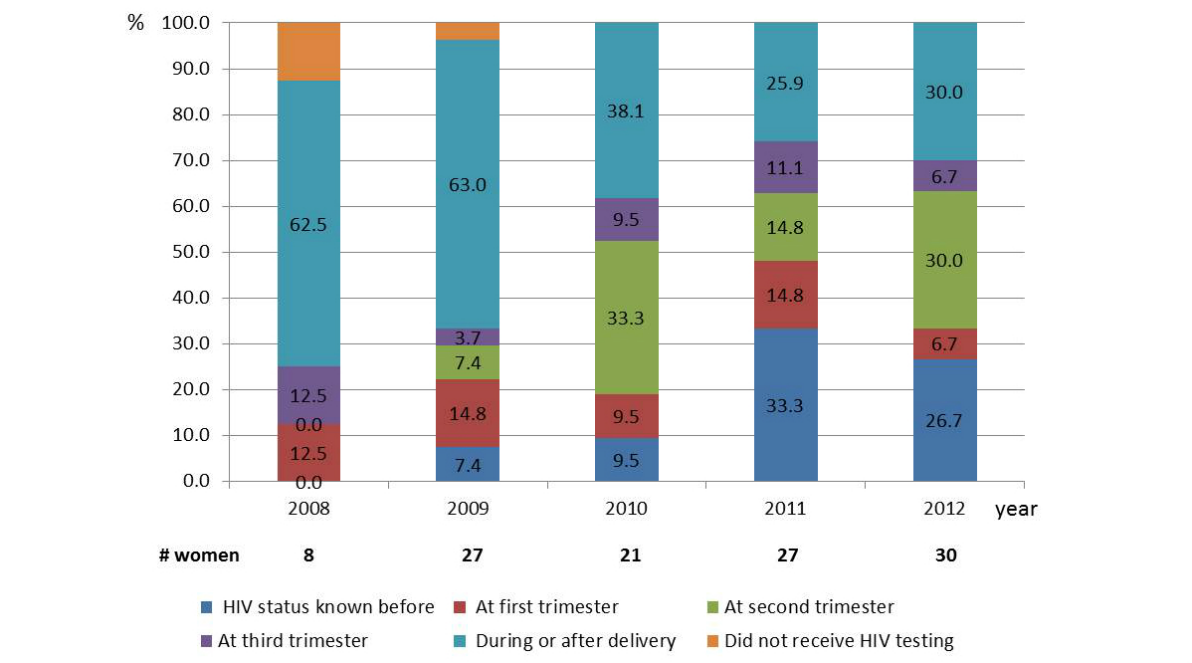
Time of HIV diagnosis among pregnant women living with HIV by year.

In 2008, 5 out of 8 (62%) HIV-positive pregnant women were diagnosed at the time of labor. Following the introduction of the PMTCT program, late diagnosis declined to 30% (9/30) in 2012 (Figure 3). From 2010 onward, the proportion of pregnant women whose HIV status was known before or during pregnancy increased (Figure 3). Although in 2008 only 25% (2/8) of HIV-infected pregnant women received ARVs, this figure increased to 70% (21/30) in 2012. Infant ARV prophylaxis (single-dose NVP and 4 weeks of AZT) also increased from 36% (4/11) in 2008 to 93% (28/30) in 2012. During the study period, formula feeding was encouraged for HIV-positive mothers whenever possible. Since 2011, 100% of exposed infants were formula fed. Breastfeeding was reported for 10 infants born before 2011.

**Figure 4:**
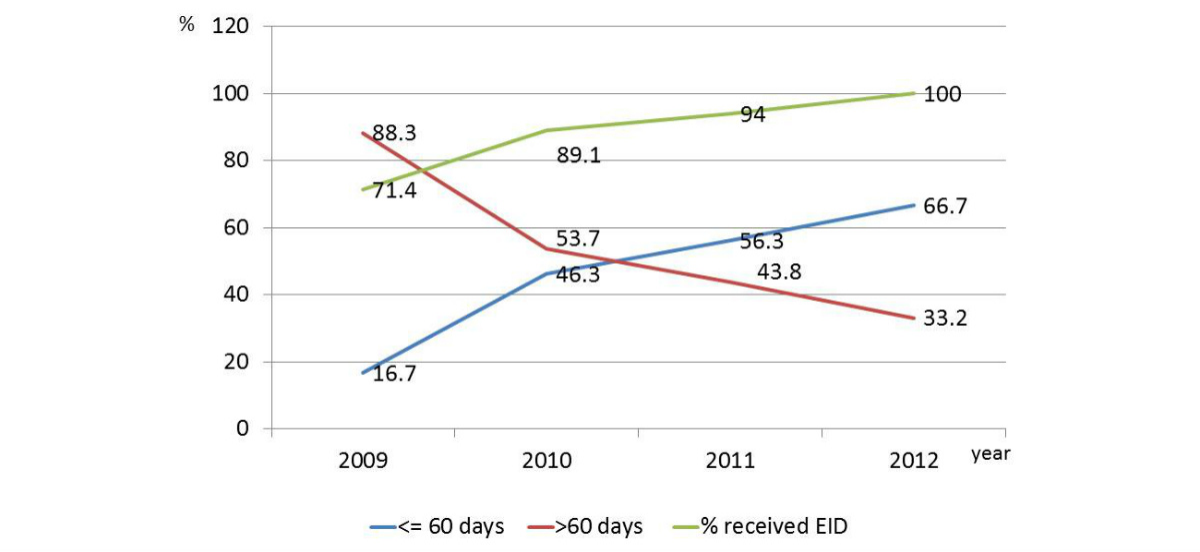
Access to early infant diagnosis (EID) by year.

The proportion of HIV-exposed infants less than 18 months of age receiving polymerase chain reaction (PCR) tests increased over time from 71.4% (30/42) in 2009 to 100% (30/30) in 2012. Notably, the proportion of infants receiving PCR tests within 60 days of birth increased from 16% (5/30) in 2009 to 67% (20/30) in 2012 (Figure 4).

### Program Limitations

Despite these improvements, there are limitations to be addressed. Late diagnosis of HIV during labor/delivery still occurred in a significant proportion of pregnant women in 2012. By the time this study concluded, only 45.4% (54/119) HIV-positive pregnant women enrolled for care and received ART. Time from delivery to enrollment in care varies from 6 days to 3 months; for 79% (43 out 54) of the women on ART, enrollment took place after 30 days following the delivery. There were 7 HIV-exposed infants documented as lost to follow-up.

### Outcomes for HIV-Exposed Infants

Data from 163 pediatric records of children born to 161 HIV-positive mothers from 2008 to 2012 showed that 84.7% infants were HIV free, 11.0% were HIV positive, and 4.3% had unknown HIV status. All 18 HIV-infected children were enrolled in care and treatment, of whom 11 received ART (1 of them died) and 7 received cotrimoxazole prophylaxis therapy for the prevention of opportunistic infections (1 of them died). Among 7 children with unknown HIV status, 2 died and 5 were lost to follow-up. The mean birth weight of infants in this cohort was 3039 g. Low birth weight (<2500 g) was observed in 3% of HIV-exposed infants.

### Transmission Rate and Risk Factors for Transmission

HIV transmission from mother to child was documented in 18 infants. The transmission rate substantially decreased from 27.3% in 2008 to 6.7% in 2012. Univariate analysis found that late HIV diagnosis for mothers increased the risk for HIV transmission to their children (odds ratio [OR] 14.7, 95% CI 1.8-121.4; *P*=.01), whereas access to ARVs for mothers (OR 0.07, 95% CI 0.008-0.6; *P*=.01), access to ARV prophylaxis for infants (OR 0.1, 95% CI 0.03-0.4; *P*=.002), and formula feeding (OR 0.3, 95% CI 0.005-0.1; *P*<.001) reduced the risk for HIV transmission from mothers to infants (Table 2). Other factors such as age and education level of mothers, delivery mode, living area, ethnicity, and employment status were not associated with HIV transmission (Table 2). We were unable to conduct multivariate analysis because of the small sample size and high collinearity among variables. Instead, we generated new variables on combined interventions to analyze their synergistic effects in preventing mother-to-child transmission. The findings show that full access to effective interventions significantly reduced the risk for the transmission (OR 0.01, 95% CI 0.001-0.1; *P*<.001), and to a lesser extent, access to either ARV for mother or infant and/or formula feeding reduced the likelihood of HIV transmission when compared with no interventions (Table 3).

### Infant Survival

**Figure 5:**
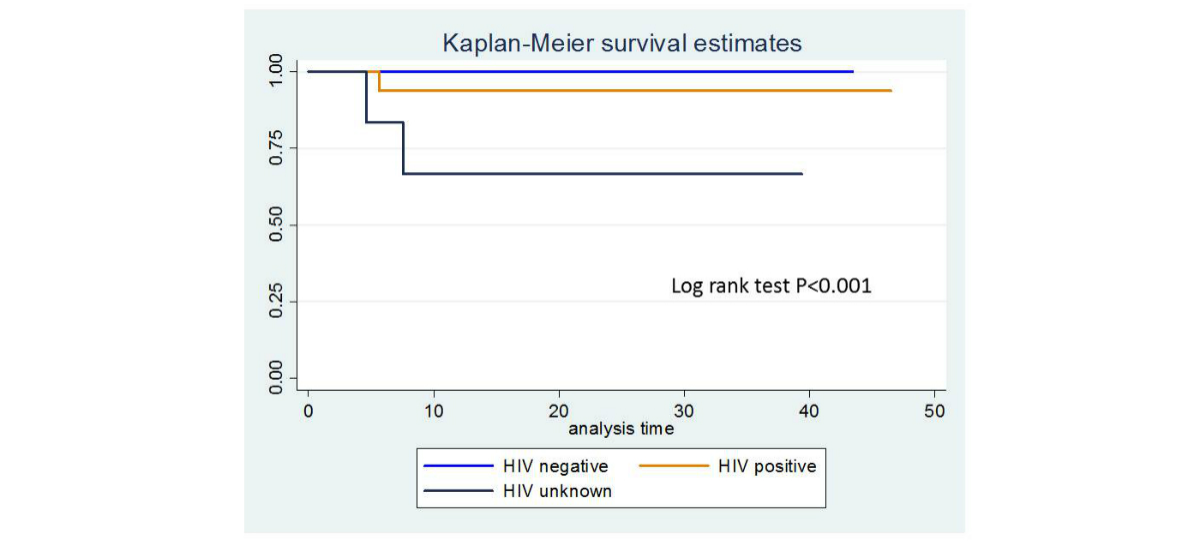
Kaplan-Meier curve for survival by accessing to intervention.

**Figure 6:**
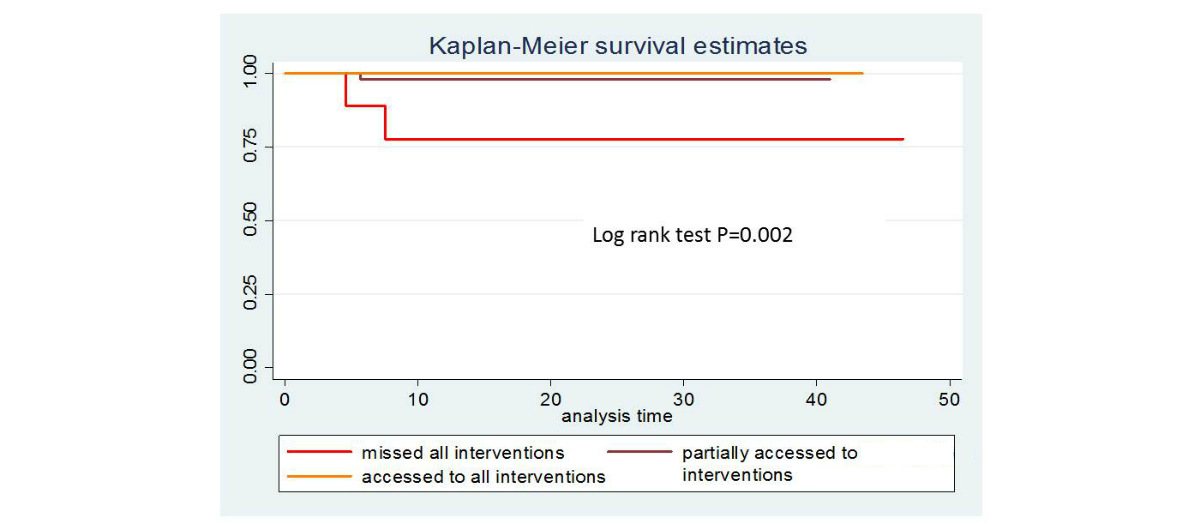
Kaplan-Meier curve for survival by accessing to intervention.

Mean age of children born to HIV-infected mothers in this study was 17 months (0.9-49 months). At 12 months, overall survival rate was 97.7%. For HIV-negative children, 12-month survival was 100%; for infants with unknown or HIV-positive status, it was 87%. High 12-month survival rates were also found among formula-fed HIV-exposed infants (98.7%). The overall infant mortality rate was 9.7/1000 live infants. Kaplan Meier survival estimates show that infants with unknown HIV status or infants who missed all interventions had lower survival rates (Figures 5 and 6). Birth weight, infant gender, and feeding mode were not associated with infant survival. Owing to small sample size, it was not possible to conduct further analysis.

**Table 2:** Univariate analysis of factors associated with infant HIV status. This table included 113 mother-baby pairs.

Variables		Infant HIV status		OR^a^ (95% CI)	*P* value
		Negative, n (%)	Positive, n (%)		
**Time of mother’s HIV diagnosis**					
	Before or during pregnancy	63 (98.4)	1 (1.6)	Referent	.01
	During or after labor/delivery	36 (80)	9 (20)	14.7 (1.8-121.4)	
**Mother received antiretroviral (ARV) during pregnancy**					
	No	41 (82)	9 (18)	Referent	.01
	Yes	62 (98.4)	1 (1.6)	0.07 (0.008-0.6)	
**Infant received ARV prophylaxis**					
	No	10 (66.7)	5 (3.3)	Referent	.002
	Yes	93 (94.9)	5 (5.1)	0.1 (0.03-0.4)	
**Infant feeding**					
	Breastfeeding	4 (40)	6 (60)	Referent	<.001
	Formula feeding	99 (96.1)	4 (3.9)	0.3 (0.005-0.1)	
**Mode of delivery**					
	Cesarean section	36 (94.7)	2 (5.3)	Referent	
	Vagina delivery	49 (94.1)	3 (5.9)	0.9 (0.1-5.7)	.90
	Unknown	18 (75.0)	5 (25)	4.5 (0.98-20.9)	.05
**Education of mother**					
	Primary/Secondary school	39 (90.7)	4 (9.3)	Referent	.60
	High school or higher	40 (87)	6 (13)	1.4 (0.4-5.3)	
**Age of mother, in years**					
	<25	26 (96.3)	1 (3.7)	Referent	.30
	≥25	9 (11.0)	3.0 (0.4-25.2)	73 (89.0)	
**Living area**					
	Urban	24 (96)	1 (4)	Referent	.30
	Rural	75 (89)	9 (11)	2.7 (0.3-22.7)	
**Ethnic minority**					
	No	77 (91.7)	7 (8.3)	Referent	.70
	Yes	26 (89.7)	3 (10.3)	1.3 (0.3-5.3)	
**Employment**					
	Having stable job	59 (90.8)	6 (9.2)	Referent	.60
	Having an unstable job or no job	44 (91.7)	4 (8.3)	0.9 (0.2-3.3)	

^a^OR: odds ratio.

**Table 3:** Multivariate analysis of combined interventions for pregnant women living with HIV infection. Interventions used were antiretrovirals (ARVs) for mothers and ARV and infant formula for infants. The table included 113 mother-baby pairs.

Variables	HIV status		OR^a^ (95% CI)	*P* value
	Negative, n (%)	Positive, n (%)		
Missed all three interventions	4 (44.4)	5 (55.6)	referent	-
Missed one or two interventions	38 (90.5)	4 (9.5)	0.08 (0.02-0.4)	.004
Full access to interventions	61 (98.4)	1 (1.6)	0.01 (0.001-0.1)	<.001

^a^OR: odds ratio.

## Discussion

### Principal Findings

The Thai Nguyen PMTCT program achieved higher coverage of ARV prophylaxis (70% vs 47.4%) and early diagnosis (66.7% vs 24.4%) than the national average in 2012 [2]. The increasing trend in access to PMTCT services, including early HIV diagnosis for mothers and infants, and ARV prophylaxis has reduced HIV infection among newborn infants from 27.3% in 2008 to 6.7% in 2012. A study that reviewed data on early infant diagnosis in 29 selected provinces in Vietnam reported the HIV transmission rate in the same period (2010-2012) to be 8.5% higher than that in Thai Nguyen [9]. We also observed a shift toward more women with a known HIV status enrolling in PMTCT program over time.

The Vietnam national response to HIV has substantially scaled up since 2005. The number of PLHIV receiving ART increased to 82,687 in 2013 (67.6% of those eligible) [2]. Similarly, the proportion of pregnant women living with HIV receiving ARV prophylaxis also increased from 13.9% in 2007 to 57% in 2013 [2]. Program monitoring and impact evaluation are moving toward increased use of program data in the interest of sustainability and more rapid assessments. Stand-alone surveillance activities are being replaced with more sustainable systems. Those systems need to be built on routinely collected program data. This community-based retrospective cohort study relied on routinely collected data augmented by surveys to illustrate that PMTCT program in Thai Nguyen significantly improved outcomes in access to HIV testing, ARV, and early infant diagnosis, thereby resulting in a reduction in HIV transmission from mother to child. The study also identified some gaps including mothers’ late enrollment to care and loss to follow-up of the infants. Factors associated with vertical HIV transmission and infant survival were examined. The risk for HIV transmission was significantly reduced if mothers accessed ARV during pregnancy and infants has access to both ARV and infant formula. Infant survival rate was significantly associated with HIV status.

WHO released *Guidelines for assessing the utility of prevention of mother-to-child transmission (PMTCT) programme data for HIV sentinel surveillance among pregnant women* in 2013 [10], followed by *Guidelines for conducting HIV surveillance based on routine programme data* in 2015 [11]. Taken together, the guidelines encourage national surveillance programs to improve PMTCT data to replace periodic ANC surveys. This study demonstrates the potential for program data to measure impact with some modifications. The lessons learned suggest additional modifications to PMTCT program data to extend the use of these data beyond standard HIV surveillance.

National data for PMTCT programs, especially capturing each step of the continuum of care cascade, is still limited because of weak or incomplete health information systems, low quality data, and challenges to link each step of the cascade. The current reporting system does not link testing and treatment/prophylaxis for pregnant women and HIV status of their infants. An important challenge encountered was the lack of a link between the different program data sources. With the absence of such a link, data validation and use to analyze PMTCT cascades is laborious and time consuming. This impedes the ability to inform national planning and policy development for prevention of new HIV infection among newborns. As recommended by WHO HIV testing guidelines [12], data from high-performing PMTCT sites that meet specified criteria can be used to replace ANC sentinel surveillance data, freeing those resources and potentially providing a more national picture than ANC-based surveys. In addition, methods used in this study can be used to measure the impact of national PMTCT programs, which is also recommended by WHO [13]. Although this study demonstrates that it is feasible to conduct retrospective cohort studies to measure impact of PMTCT programs, it would be more cost-effective and timely if a unique identifier system can link data from pregnant women living with HIV with their infants’ health records to minimize time and cost for linking and validating mother-baby pairs and to facilitate tracking and follow-up of women and their HIV-exposed infants.

The issue of enrollment of HIV-infected pregnant women in OPCs at a late stage of infection remains. In this study, only 62% of pregnant women living with HIV attended an OPC for care and treatment (excluding 10 women who terminated their pregnancies or died). Some women enrolled for care and treatment several months after delivery. This suggests poor linkages between maternal and child health services and HIV services. A functioning linkage from testing to enrollment for PMTCT and treatment and care requires strong and effective collaboration between reproductive health and HIV programs. Currently, Vietnam has adopted WHO 2016 recommendations [14] on treating all HIV-infected pregnant women, which could facilitate the enrollment in care and minimize loss to follow-up. Linkages can improve overall care and retention; Cambodia uses linkages and a strong referral and follow-up system to increase uptake of HIV testing among pregnant women and link with PMTCT services [15].

Although HIV transmission in Thai Nguyen has been reduced remarkably, it is still above 5%. To achieve the goal of elimination of mother-to-child transmission, it is important to understand factors associated with HIV transmission. Late maternal HIV diagnosis, high maternal viral load and low CD4 count, breastfeeding, vaginal delivery, and invasive procedure are documented risk factors for mother-to-child HIV transmission [16-20]. In our univariate analysis, education level, ethnicity, living area, and employment status were not associated with infant HIV status, although a study in Vietnam found an association between young age of the mothers (<25) and HIV transmission [21]. It is important to note that key effective interventions, including time of early HIV diagnosis, access to ARVs for mothers and infants, and infant formula feeding, were associated with low risk for HIV transmission. The full access to combined interventions of ARV for mothers and infants and formula feeding for infants has significantly reduced the risk for HIV transmission from mother to child. This finding suggests that a universal HIV-testing strategy should be implemented to achieve the national target of elimination of HIV transmission from mother to child by 2030. Universal testing may require initial investments, but it has been shown to be cost-effective even in a low and concentrated HIV epidemic country such as Vietnam [22].

The ultimate goal of PMTCT is to save the lives of infants born to HIV-positive mothers. The impact of the PMTCT program could be measured in terms of lives saved/infections averted. The Thai Nguyen study showed higher survival rate among HIV-negative infants (100%) compared with HIV-positive or unknown HIV-status infants (87%) at 12 months of age. This is consistent with another study from Vietnam [21], which demonstrated that no HIV-negative infants died in the first 12 months of their lives. Factors that increased mortality risk among HIV-exposed infants reported in several studies [23-26] were maternal death, maternal high viral load and low CD4 count, and infant HIV infection.

### Limitations

There are several limitations in this study. A small number of mother-child pairs could not be found according to the address given at clinics, which may introduce biases to our understanding of program effectiveness, although without knowing the status of the women who were lost, it is difficult to assess. In addition, the data were based on program data and therefore did not include those who were not diagnosed or not reported; thus, results might have been underestimated. Recall bias may also have occurred as mothers were asked to report on events that happened in the past such as time of first ANC visits and time of HIV diagnosis. The analysis of factors associated with mother-to-child transmission of HIV was limited because of small sample size and lack of data on psychological, social support, and biological variables such as mothers’ CD4 count and viral load. Finally, improvement of the PMTCT outcomes might have been influenced by other factors, including expansion of the national ART program, which could not be determined in this study.

### Conclusions

This study demonstrated that modifications to routine data collection systems can yield data to evaluate the impact of PMTCT programs. A routine data-based surveillance system derived from PMTCT program data can be established to provide rich information on programs’ challenges and successes. Significant improvement in access to HIV services and prevention of HIV transmission from mother to child was documented in Thai Nguyen province. However, factors that increased the risk of HIV transmission, including late diagnosis and late enrollment for care, remain to be addressed. In addition to expanding current harm reduction programs to reduce the rate of new infection among PWID and their female partners, enhancements to facilitate better uptake of ANC, identification of HIV-infected pregnant women, and their linkage to care are still needed. Increased access to CD4 and viral load testing, as well as steps to reduce loss to follow-up will also improve outcomes.

To achieve the national goal of elimination of mother-to-child transmission by 2020, greater efforts are needed to ensure that pregnant women know their HIV status earlier, preferably in the first trimester. Considering this, integration of HIV testing as a part of ANC package is expected to improve coverage of HIV testing for pregnant women. Finally, a strong information management system linking HIV and maternal and child health programs is required to ensure sustainable ongoing measurement of the impact of PMTCT programs to better serve pregnant women living with HIV and their infants with appropriate treatment.

## Abbreviations

AIDS: acquired immunodeficiency syndrome
ANC: antenatal care
ART: antiretroviral therapy
ARV: antiretroviral
AZT: zidovudine
CD4: cluster of differentiation 4
FSW: female sex workers
MSM: men who have sex with men
NVP: nevirapine
PLHIV: people living with HIV
PMTCT: prevention of mother-to-child transmission of HIV
PW: pregnant women
PWID: people who inject drugs
OPC: outpatient clinic
OR: odds ratio
UNAIDS: Joint United Nations Program on HIV and AIDS
WHO: World Health Organization
